# Cloning, Purification and Characterization of the Collagenase ColA Expressed by *Bacillus cereus* ATCC 14579

**DOI:** 10.1371/journal.pone.0162433

**Published:** 2016-09-02

**Authors:** Carmen M. Abfalter, Esther Schönauer, Karthe Ponnuraj, Markus Huemer, Gabriele Gadermaier, Christof Regl, Peter Briza, Fatima Ferreira, Christian G. Huber, Hans Brandstetter, Gernot Posselt, Silja Wessler

**Affiliations:** 1 Department of Molecular Biology, Division of Microbiology, Paris-Lodron University of Salzburg, Salzburg, Austria; 2 Department of Molecular Biology, Division of Structural Biology, Paris-Lodron University of Salzburg, Salzburg, Austria; 3 Centre of Advanced Study in Crystallography and Biophysics, University of Madras, Guindy Campus, Chennai, India; 4 Department of Molecular Biology, Division of Allergy and Immunology, Paris-Lodron University of Salzburg, Salzburg, Austria; 5 Department of Molecular Biology, Division of Chemistry and Bioanalytics, Paris-Lodron University of Salzburg, Salzburg, Austria; Nagoya University, JAPAN

## Abstract

Bacterial collagenases differ considerably in their structure and functions. The collagenases ColH and ColG from *Clostridium histolyticum* and ColA expressed by *Clostridium perfringens* are well-characterized collagenases that cleave triple-helical collagen, which were therefore termed as ´true´ collagenases. ColA from *Bacillus cereus* (*B*. *cereus*) has been added to the collection of true collagenases. However, the molecular characteristics of *B*. *cereus* ColA are less understood. In this study, we identified ColA as a secreted true collagenase from *B*. *cereus* ATCC 14579, which is transcriptionally controlled by the regulon phospholipase C regulator (PlcR). *B*. *cereus* ATCC 14579 ColA was cloned to express recombinant wildtype ColA (ColA^wt^) and mutated to a proteolytically inactive (ColA^E501A^) version. Recombinant ColA^wt^ was tested for gelatinolytic and collagenolytic activities and ColA^E501A^ was used for the production of a polyclonal anti-ColA antibody. Comparison of ColA^wt^ activity with homologous proteases in additional strains of *B*. *cereus sensu lato* (*B*. *cereus s*.*l*.) and related clostridial collagenases revealed that *B*. *cereus* ATCC 14579 ColA is a highly active peptidolytic and collagenolytic protease. These findings could lead to a deeper insight into the function and mechanism of bacterial collagenases which are used in medical and biotechnological applications.

## Introduction

Collagen is the most abundant component of the extracellular matrix (ECM) in vertebrates, which provides not only a flexible scaffold for embedded cells, but also regulates crucially important cellular processes including differentiation, cellular growth, survival, migration, and many more [1]. Dynamic remodeling of the ECM constantly requires redistributions, modifications and also degradation of ECM components to maintain functional tissue architecture [1, 2]. Numerous collagenases have been described as non-specific or pseudocollagenases. Pseudocollagenases degrade gelatin or non-helical regions of collagen, while only true collagenases can cleave triple-helical regions within the three chains of native collagen [3]. Both types of proteases are strongly associated with diseases like metastasis of tumors, inflammation, ulceration, rheumatoid arthritis or bacterial infections [4]. Examples for pseudocollagenases are mammalian tissue enzymes like pepsin, trypsin, chymotrypsin or papain. The group of true collagenases includes selected members of the matrix metalloprotease family (MMP-1, -8, -13, -14) and cathepsin K which contribute to ECM proteolysis [5, 6]. Additionally, bacterial collagenases can interfere with collagen functions in the ECM. Pathogens such as *Borrelia burgdorferi*, *Peptostreptococcus magnus*, *Vibrio alginolyticus*, *Achromobacter lyticus*, *Porphyromonas gingivalis*, *Treponema denticola*, *Streptococcus gordnii*, *etc*. express collagenases belonging to the group of metalloproteases, serine proteases or thiol proteases [4]. In particular, collagenases expressed by *Clostridium histolyticum* constitute well characterized paradigms which are, together with additional proteases and toxins, implicated in clostridial-dependent myonecrosis [7, 8].

Clostridial ColH and ColG have been described as zinc-dependent metalloproteases that contain an N-terminal signal peptide, a putative propeptide, an activator domain followed by the catalytic peptidase domain, one or two polycystic kidney disease-like domain (PKD) domains, and one to three collagen binding domains (CBD) [8, 9]. As members of the gluzincin subfamily, clostridial collagenases bind the catalytic zinc ion via the two histidine residues in the consensus HEXXH sequence in the active center; a third zinc ligand is provided by a glutamate approximately 33–35 amino acids downstream of the HEXXH motif [10]. Structural data are available for the catalytic domains of ColG and ColH from *C*. *histolyticum* and ColT from *C*. *tetani* revealing a double glycine motif upstream of the HEXXH motif, which is also necessary for the collagenase activity [11, 12]. Upon calcium binding the PKD-like domain of ColG undergoes a conformational domain rearrangement [9, 13]. Both, zinc and calcium binding is required for full proteolytic activity [9]. In a proposed two-state model of ColG, collagen recognition, binding and cleavage involve an opened and closed ColG conformation [13], indicating a coordinated mechanism in collagenase function. The preferred cleavage sites cover the typical collagen motifs Gly-Pro-X and Gly-X-Hyp (hydroxyproline) [14].

Besides Clostridia, collagenolytic and gelatinolytic activities have also been observed in several bacterial species of *Bacillus cereus s*.*l*. (i.e. *B*. *cereus sensu stricto* [*B*. *cereus s*.*s*.], *B*. *mycoides*, *B*. *pseudomycoides*, *B*. *thuringiensis*, *B*. *weihenstephanensis*, *B*. *anthracis* and *B*. *cytotoxicus* [15]) [16–18], which are less characterized. *B*. *cereus s*.*s*. and its closely related family members are Gram-positive, spore forming and facultative anaerobic bacteria ubiquitously found in the environment. As an opportunistic bacterium, *B*. *cereus s*.*s*. has been identified as an increasing cause of food-borne diseases leading to gastrointestinal disorders, but also of non-gastrointestinal diseases in patients [19–22]. *B*. *cereus* pathogenicity is associated with the expression of several toxins and virulence factors. Among them, hemolysins, phospholipase C, emetic toxin, proteases or enterotoxins have been connected to the induction of gastrointestinal and non-gastrointestinal diseases, such as endophthalmitis [23, 24], wound infections [25, 26] or rare cases of postoperative meningitis [27, 28] and pneumonia [29, 30]. Most of these factors are regulated by the pleiotropic regulon phospholipase C regulator (PlcR) [31]. PlcR-regulates gene expression via a conserved palindromic sequence in the promotor regions of its target genes [32, 33]. *B*. *cereus* also expresses collagenases in a PlcR-dependent manner [31], which might contribute to bacterial pathogenesis through the degradation of collagen in the ECM. For instance, it was suggested that collagenases facilitate *B*. *cereus* invasion into the eye lens and thus promote bacterial endophthalmitis [34].

Studies on *B*. *cereus* collagenase activity were exclusively performed using secreted collagenase(s) enriched from supernatants of bacterial liquid cultures [17, 18]. Comparable to clostridial collagenases, it was demonstrated that *B*. *cereus* collagenase activity is zinc-dependent, could be induced by calcium ions [17], and targets native collagen [17, 18]. However, detailed biochemical analyses have not been performed so far.

In a previous study, we have detected gelatinolytic activities of *B*. *cereus* ATCC 14579 in zymography analyses and which are strongly active in the logarithmic growth phase [35]. To obtain deeper insights into the molecular functions of the collagenolytic *B*. *cereus* proteases, we identified the endogenously expressed collagenase and analyzed recombinant ColA acting as a highly active and secreted true collagenase from *B*. *cereus* ATCC 14579.

## Materials and Methods

### Bacteria

*Bacillus cereus* (ATCC 14579) and *Bacillus thuringiensis* (strain 407) were obtained from Nalini Ramarao (INRA, La Minière, Guyancourt, France). The *B*. *cereus* ATCC 14579 deletion mutant lacking the *plcR* regulon (BcΔ*plcR*) was a kind gift from Michel Gohar (INRA, Génétique Microbienne et Environnement Jouy-en-Josas, France). *Bacillus subtilis* (DSM 402), *Bacillus weihenstephanensis* and *Bacillus megaterium* were included as further controls. All bacteria were grown in brain heart infusion (BHI) medium (Sigma Aldrich) at 37°C overnight shaking at 200 rpm. Overnight cultures were diluted 1:20 in fresh BHI medium and bacteria and their corresponding supernatants from the liquid cultures were harvested after indicated time periods by centrifugation at 3000 x g for 10 min at 4°C. Supernatants were sterile-filtered (0.22 nm filter, Greiner). Bacterial pellets were resuspended in lysis buffer (20 mM Tris pH 7.5, 100 mM NaCl, 1% Triton X-100, 0.5% DOC, 0.1% SDS, 0.5% NP-40) and sonicated 3 x for 30 sec with 50% power [35]. Bacterial lysates were cleared from debris by centrifugation at 16000 x g for 10 min at 4°C and protein amounts were determined by Bradford protein assay (Carl Roth).

### Mass-Spectrometry

Supernatants of *B*. *cereus* ATCC 14579 were loaded on a preparative gelatin zymogram as described before [35]. Briefly, transparent bands were excised from the zymogram and proteins were electro-eluted using D-Tube^™^ Dialyzer Midi (Merck Millipore). After concentrating using Centricon^®^ (Merck Millipore), the protein samples were separated via SDS-PAGE. To visualize the proteins, gels were stained using 1% Coomassie Brilliant Blue G250 (BioRad). Protein bands were excised and digested with the ProteoExtract All-in-One Trypsin Digestion Kit (Merck Millipore). Resulting peptides were separated by reverse-phase nano-HPLC (Dionex Ultimate 3000, Thermo Fisher Scientific). Peptides were loaded onto the trap column (PepSwift Monolithic Trap Column, Dionex) and desalted with 0.1% (v/v) heptafluorobutyric acid at a flow rate of 10 μl/min. After 5 minutes, trap and separation column (PepSwift Monolithic Nano Column, 100 μm x 25 cm, Dionex) were coupled with a switching valve and the peptides were eluted with an acetonitrile gradient (Solvent A: 0.1% (v/v) FA/0.01% (v/v) TFA/5% (v/v) ACN; solvent B: 0.1% (v/v) FA/0.01% (v/v) TFA/90% (v/v) ACN; 5–45% B in 60 min) at flow rate of 1 μl/min at 55°C. The HPLC was directly coupled via nano electrospray to a Q-Exactive Orbitrap mass spectrometer (Thermo Fisher Scientific). Capillary voltage was 2 kV. For peptide identification, a top 12 method was used with the normalized fragmentation energy at 27%. For protein identification, Proteome Discoverer version 1.4 (Thermo Fisher Scientific) with SequestHD and UniProtKB was used.

To analyze the cleavage site of the GST-ColA ΔSP, the GST fragment was precipitated using glutathione sepharose. After elution, the samples were diluted in 0.1% (v/v) aqueous formic acid to a final concentration of 0.1 mg/ml. Intra-molecular disulfide bonds were reduced with 5 mM TCEP (Sigma-Aldrich Chemie GmbH) at 60°C for 30 minutes. The analysis was carried out on a high-performance liquid chromatography (HPLC) system (Ultimate 3000, Thermo Fisher Scientific) at a flow rate of 200 μl/min. A Supelco Discovery C18 column (150 × 2.1 mm i.d., 3 μm particle size, 300 Å pore size, Sigma-Aldrich) was operated at a column temperature of 50°C. 10 μl of sample were injected in in-line split-loop mode. Separation was carried out with a gradient of 5.0–50.0% (v/v) acetonitrile (ACN, VWR) in 0.1% (v/v) formic acid (FA, Sigma-Aldrich Chemie GmbH) in 20 min, followed by column regeneration at 99.99% ACN in 0.1% FA for 10 min and re-equilibration at 5.0% in 0.1% FA for 15 min. UV-detection was carried out with a 2.5 μl flow-cell at 214 nm. Mass spectrometry was conducted on a quadrupole-Orbitrap instrument (QExactive) equipped with an Ion Max source with a heated electrospray ionization (HESI) probe from Thermo Fisher Scientific. Mass calibration of the instrument was conducted with Pierce^™^ LTQ Velos ESI Positive Ion Calibration Solution from Life Technologies. The instrument settings were as follows: source heater temperature of 200°C, spray voltage of 4.0 kV, sheath gas flow of 15 arbitrary units, auxiliary gas flow of 5 arbitrary units, capillary temperature of 300°C, S-lens RF level of 60.0, in-source CID of 20.0 eV, AGC target of 1e6 and a maximum injection time of 200 ms. The intact protein measurements were carried out in full scan mode with a range of m/z 1,000–2,500 at a resolution of 140,000 at m/z 200. Deconvolution of the intact ion spectra was carried out with the Xtract algorithm integrated into the software Xcalibur 3.0.63 (Thermo Fisher Scientific).

### Cloning, Mutagenesis and Purification of ColA

The collagenase ColA (BC3161, Gene ID: 1205508) was amplified from bacterial genomic DNA from *B*. *cereus* strain ATCC 14579. PCR primers (Table 1) were designed to amplify ColA lacking the predicted signal peptide (ColA ΔSP, aa 31–960) or lacking the predicted propeptide (ColA ΔPP, aa 93–960). The amplified *Bam*HI/*Xho*I flanked PCR products were cloned into the pGEX-6P-1 plasmid (GE Healthcare Life Sciences) and transformed in *E*. *coli* BL21 to create a GST-ColA fusion protein. To generate a protease-inactive ColA protein, glutamic acid 501 was substituted by an alanine (ColA^E501A^) (primers in Table 1) using the QuikChange Lightning Site-Directed Mutagenesis Kit (Agilent) according to the manufacturer’s instructions. For heterologous expression and purification of ColA proteins, transformed *E*. *coli* was grown in 300 ml LB medium to an OD_600_ of 0.5–0.7 at 37°C and 200 rpm and the expression was induced by the addition of 0.1 mM isopropylthiogalactosid (IPTG) at 30°C for 3 h. The bacterial culture was pelleted at 4500 x g for 30 minutes and bacteria were lysed in 10 ml ice-cold PBS by sonication. The lysate was cleared by centrifugation and the supernatant was incubated with glutathione sepharose (GE Healthcare Life Sciences) at 4°C overnight as described earlier [36]. The fusion protein was either eluted with 10 mM reduced glutathione for 10 minutes at room temperature or cleaved with 180 U Prescission Protease for 16 h at 4°C (GE Healthcare Life Sciences).

**Table 1:** Primer used in this study.

Pathogen	Acc.no^a^	Protein	PCR primer^b^
*Bacillus cereus 14579*	BC3161	ColA ΔSP	f: 5’-GATCGGATCCGAAGAAACAGCACCCTATAATATC-3’
			r: 5’-CATGCTCGAGTCATTTTACTAATAATGAATATTC-3’
		ColA ΔPP	f: 5’-GATCGGATCCTATACTTTGGCAGAACTGAATAAAA-3’
			r: 5’-CATGCTCGAGTCATTTTACTAATAATGAATATTC-3’
		ColA E501A	**Mutagenesis primer**^c^
			f: 5’-GAGTTATTCCGTCATGCATTCACTCATTATTTAC-3’
			r: 5’-GTAAATAATGAGTGAATGCATGACGGAATAACTC-3’

^a^ accession number;

^b^ restriction recognition sites are underlined;

^c^ substituted nucleotides are underlined

### Zymography

Bacterial lysates, supernatants or recombinant proteins were separated by SDS-PAGE containing 0.1% gelatin under non reducing conditions as described previously [36]. Proteins separated by the gels were renatured (2.5% TritonX-100) for 1 h, incubated in developing buffer (50 mM Tris pH 7.5, 200 mM NaCl, 5 mM CaCl_2_, 0.02% Brij35) for 16 hours at 37°C and stained using 0.5% Coomassie Brilliant Blue R250 (BioRad).

### SDS-PAGE and Western Blot

Proteins were separated by SDS-PAGE under reducing conditions and stained using 1% Coomassie Brilliant Blue G250 (BioRad). For Western blot analyses, equal amounts of proteins were separated by SDS-PAGE and blotted on PVDF membranes. *B*. *cereus* ATCC 14579 ColA was detected using a polyclonal anti-ColA antiserum produced in rabbits immunized with recombinant ColA ΔPP^E501A^ (Davids Biotechnology, Germany). To detect the GST-tag, an anti-GST antibody (Rockland) was applied. Visualizing was performed using Odyssey^®^ Fc Imaging System (LiCor).

### *In Vitro* Cleavage Assays

Enzymatic assays with N-[3-(2-Furyl)acryloyl]-Leu-Gly-Pro-Ala (FALGPA) as substrate were performed as described by van Wart and Steinbrink [37] and detailed in the manufacturer’s protocol (AppliChem) with some minor modifications. Briefly, ColA ΔPP^wt^ or ColA ΔPP^E501A^ were incubated with FALGPA in 250 mM Hepes, 10 mM CaCl_2_, 10 mM ZnCl_2_. Collagenase G from *Clostridium histolyticum* (ColG) (Q9X721; Y^119^-K^1118^) and the peptidase domain of *C*. *tetani* ColT (ColT^PD^) (Q899Y1, D^340^-K^731^) were used as controls [9]. The enzymes were analyzed at 0.67 μM using a substrate concentration of 2 mM FALPGA in 60 μL reaction volume. The concentration of FALGPA was verified in solution via UV absorbance at 305 nm (ε_305_ = 24.70 mM^-1^ cm^-1^). All measurements were performed at 25°C, and the decrease in absorbance upon substrate cleavage was monitored at 345 nm in 1 min intervals for 25 min with an Infinite M200 plate reader (Tecan). Complete substrate turnover (100%) was accomplished by ColT^PD^ and ColA ΔPP^wt^ after 20 min. All experiments were performed in triplicates and repeated three times. Ratio of substrate turnover (+/- SD) was calculated normalized on the lowest substrate absorbance measured. Significance was calculated using Student´s t-test (paired, one tailed) with a significance threshold of p < 0.05 (GraphPad Prism 5).

For the collagenolytic assays, pepsin-extracted soluble type I collagen from bovine skin (Cell Guidance Systems Ltd) was used at a final concentration of 1 mg/ml (*i*.*e*. 3.33 μM assuming a molecular mass of 300 kDa, corresponding to non-polymerized tropocollagen) in 250 mM Hepes pH 7.5, 150 mM sodium chloride, 5 mM calcium chloride and 5 μM zinc chloride. The collagen was incubated with 0.83 μM alpha-chymotrypsin (Sigma-Aldrich) and 0.055 μM ColG, ColT^PD^, ColA ΔPP^wt^ and ColA ΔPP^E501A^ at 25°C. Samples were taken at the indicated time points and the reactions were stopped with 10 mM copper chloride for alpha-chymotrypsin, and with 50 mM EDTA for the other proteins. To test whether ColA targets additional proteins from the ECM, 1 μg ColA ΔPP was incubated with 1 μg fibrinogen (Bovine Plasma, Calbiochem), 1 μg human vitronectin (R&D), 1 μg laminin (mouse, BD Bioscience), 1 μg collagen (Sigma) or 5 μg casein (Carl Roth) in 50 mM HEPES pH 7.4 or collagenase cleavage buffer containing 50 mM Tris, 500 mM NaCl, 20 mM CaCl_2_, pH 7.6 for 16 hours at 37°C.

### Homology Model

The full length amino acid sequence of *B*. *cereus* ATCC 14579 ColA (Q81BJ6) was used as a target sequence to search for templates using BLASTp against the protein data bank (PDB) structures [38] as well as the SWISS-MODEL server [39]. Among the templates obtained from the SWISS-MODEL, the 2.5 Å crystal structure of apo collagenase G (ColG) from *C*. *histolyticum* (PDB 2Y3U) was considered as the most suitable candidate to model the residues Y93-K850 of *B*. *cereus* ATCC 14579 ColA. A sequence identity of 49.80% was observed between the corresponding regions of *B*. *cereus* ATCC 14579 ColA and *C*. *histolyticum* ColG proteins. The quality of the obtained model (Y93-K850) was evaluated by RAMPAGE [40]. The electrostatic potential surface of both ColA and ColG was generated using the program PyMOL (The PyMOL Molecular Graphics System, Version 1.7.4 Schrodinger, LLC).

## Results

### Bacterial Species of *Bacillus cereus s*.*l*. Express Gelatinolytic Proteases

**Fig 1:**
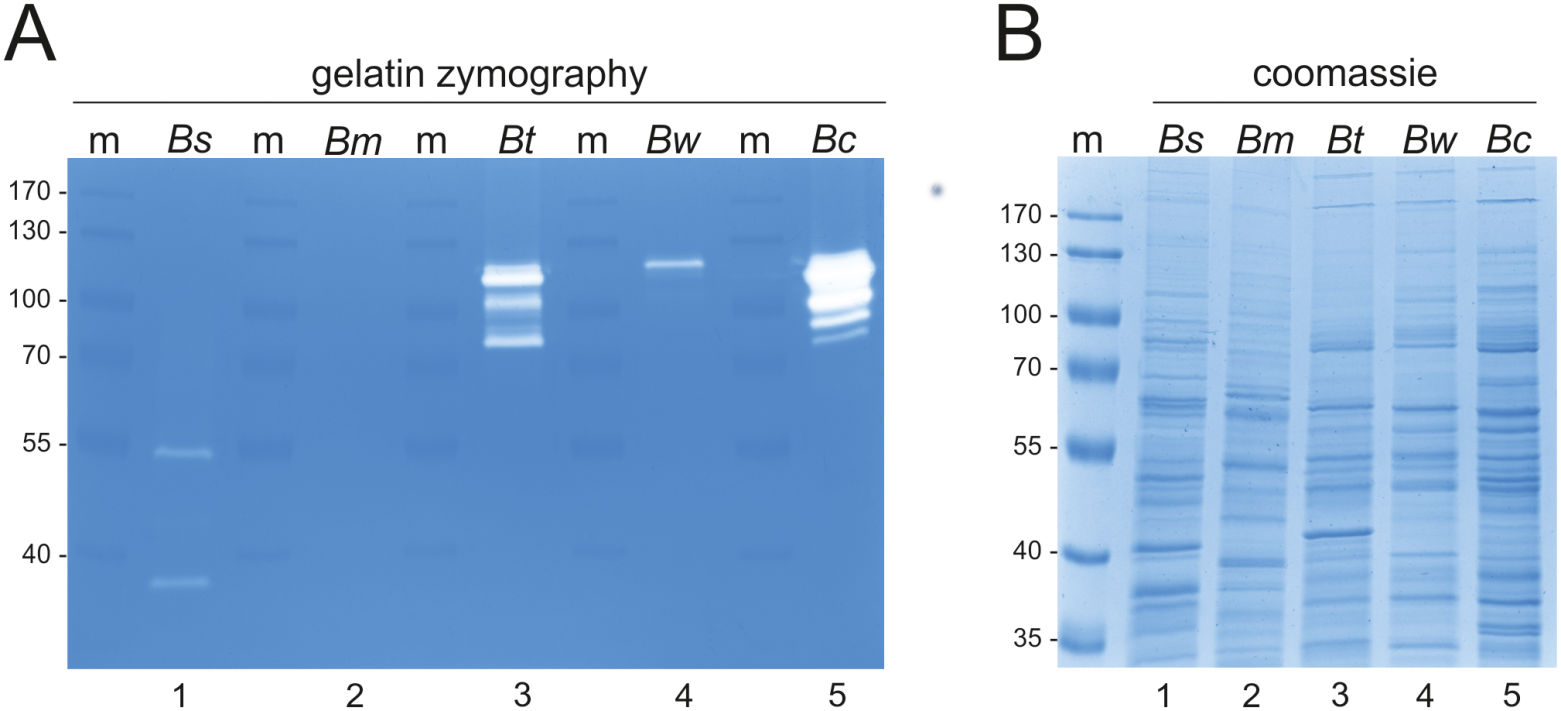
Gelatinolytic activities expressed by different *Bacillus* species. (**A**) Equal amounts of proteins in lysates of *B*. *subtilis (Bs)*, *B*. *megaterium (Bm)*, *B*. *thuringiensis (Bt)*, *B*. *weihenstephanensis (Bw)* and *B*. *cereus* ATCC 14579 *(Bc)* were analyzed for proteolytic activity in gelatin zymography. Protein standard (m) indicated molecular weights of gelatinolytic activities. (**B**) Efficient disruption of bacteria and equal protein amounts were demonstrated by coomassie-stained SDS PAGEs. Protein standard (m) indicated molecular weights of proteins.

In a previous study, we detected a gelatinolytic protease in *B*. *cereus* ATCC 14579 [35]. To analyze whether also other species from the *B*. *cereus s*.*l*. group and additional *Bacillus* species express similar active proteases, gelatinolytic activities in lysates of *B*. *subtilis*, *B*. *megaterium*, *B*. *thuringiensis* and *B*. *weihenstephanensis* were compared with *B*. *cereus* ATCC 14579 in gelatin zymography. Equal protein amounts of *B*. *subtilis (Bs)*, *B*. *megaterium (Bm)*, *B*. *thuringiensis (Bt)*, *B*. *weihenstephanensis (Bw)* and *B*. *cereus* ATCC 14579 *(Bc)* lysates were separated in a gelatin zymogram (Fig 1A). Transparent bands in the coomassie-stained gel refer to an active protease cleaving gelatin. As described previously [35], we detected active proteases in a *B*. *cereus* ATCC 14579 lysate with molecular weights between 80 kDa and 110 kDa (Fig 1A, lane 5). A similar protease pattern was also identified in a lysate of *B*. *thuringiensis* (Fig 1A, lane 3). In the lysate of *B*. *weihenstephanensis*, we observed a predominant proteolytic activity at a molecular weight of 120 kDa (Fig 1A, lane 4). In contrast, the lysate of *B*. *subtilis* showed a different activity pattern with two distinct bands at 55 kDa and 35 kDa (Fig 1A, lane 1). In *B*. *megaterium*, no activity was detectable (Fig 1A, lane 2). In order to control equal protein yield of the bacterial cultures, all lysates were analyzed by SDS PAGE and stained with coomassie brilliant blue (Fig 1B). To identify the gelatinolytic protease of *B*. *cereus* ATCC 14579, we performed a preparative zymogram, electro-eluted the proteins from the excised gel and analyzed the proteins by coomassie-stained SDS PAGEs. Excised proteins were finally examined by mass-spectrometry. Among several protein hits, two peptides of the collagenase ColT (bcere0001_4490) from *B*. *cereus* m1293 were identified that correspond to the orthologous collagenase ColA (BC_3161) from *B*. *cereus* strain ATCC 14579 exhibiting 76.8% identity (Table 2) in the N-terminus (S1 Fig).

**Table 2:** Collection of annotated collagenases in *Bacillus* strains blasted against Q81BJ6 (ColA) as protein query.

Acc.no^a^	Organism	Strain	Gene name	Protein name	Identity
Q81BJ6	*B*. *cereus*	ATCC 14579	BC3161	Microbial collagenase	100.0%
C2MFV5	*B*. *cereus*	m1293	bcere0001_4490	Collagenase ColT	76.8%
A0A0N7JLG3	*B*. *thuringiensis*	XL6	BTXL6_25515	Collagenase	98.4%
A0A0E0W2W4	*B*. *anthracis*	H9401	H9401_3141	Microbial collagenase	96.0%
A0A090YV35	*B*. *mycoides*	BHP	DJ93_3050	Collagenase family protein	74.9%
A7GL33	*B*. *cxytotoxicus*	DSM 22905	Bcer98_0486	Microbial collagenase	73.5%
C3BGN8	*B*. *pseudomycoides*	DSM 12442	bpmyx0001_8100	Microbial collagenase	73.3%
A0A0A0WXL8	*B*. *weihenstephanensis*	WSBC 10204	colA1	Microbial collagenase	72.7%

^a^ UniProt accession number

### Cloning and Purification of Recombinant *B*. *cereus* ATCC 14579 ColA

The domain structure of ColG from *C*. *histolyticum* covers an activator domain, peptidase domain, a PKD domain, and the two CBD domains alpha and beta. In comparison to ColG, clostridial ColH harbors a second PKD domain, but only one CBD domain [9, 10]. Alignment of *B*. *cereus* ColA (Q81BJ6, gene name: BC_3161) with ColH (Q46085) and ColG (Q9X721) revealed a slightly higher identity of ColA to ColG (44.77%) than to ColH (44.15%) (S2 Fig).

**Fig 2:**
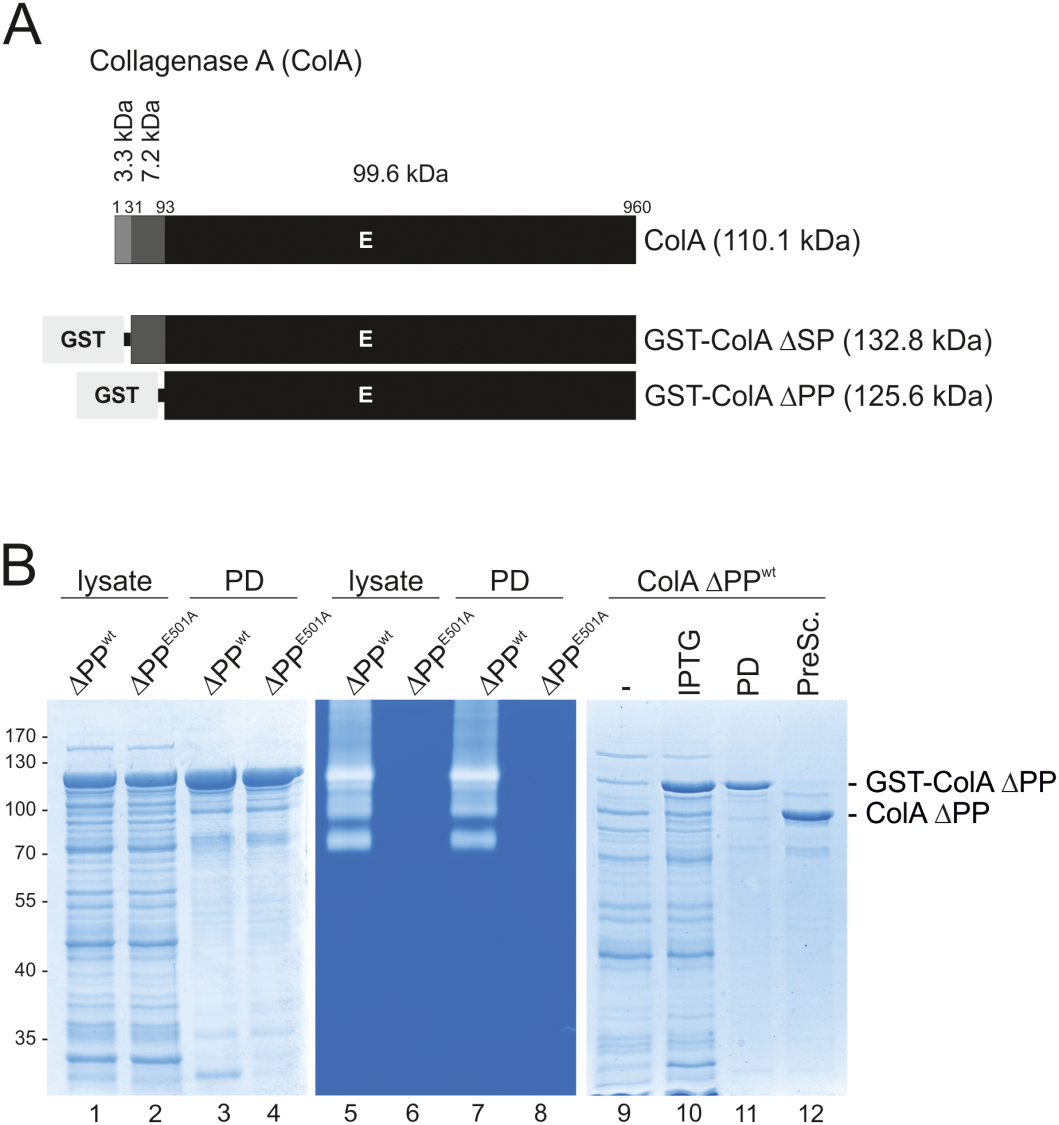
Cloning, overexpression and activity of *B*. *cereus* ATCC 14579 ColA. (**A**) ColA is expressed as a 110.1 kDa protein and consists of a 3.3 kDa signal peptide (aa 1–30), a 7.2 kDa propetide (aa 31–92) and a 99.6 kDa C-terminal part (aa 93–960) of ColA. Expression constructs for N-terminally GST-tagged ColA ΔSP (132.8 kDa) and ColA ΔPP (125.6 kDa) were cloned. Glutamic acid (E) 501 in the active center of ColA was exchanged by an alanine (E501A) to create proteolytically inactive ColA. (**B**) The expression, enrichment and activity of GST-ColA ΔPP^wt^ and GST-ColA ΔPP^E501A^ proteins in IPTG-induced *E*. *coli* lysates or purified via GST pull down (PD) experiments were analyzed by SDS-PAGE (left panel) and gelatin zymography (middle panel). To purify ColA ΔPP^wt^, transformed *E*. *coli* (-) were induced by IPTG to stimulate GST-ColA ΔPP^wt^ expression. After lysing bacteria, GST-ColA ΔPP^wt^ was bound to GST sepharose and either eluted by glutathione (PD) as a GST fusion protein (GST-ColA ΔPP^wt^) or cleaved and eluted with the PreScission protease (PreSc) to obtain the untagged protease ColA ΔPP^wt^.

Web based resources SMART (simple modular architecture research tool, http://smart.embl.de/) and SignalP (SignalP4.1, http://www.cbs.dtu.dk/services/SignalP/) [41–43] predicted a signal peptide (aa 1–30) and a propeptide in the N-terminus of ColA (aa 31–92) (Figs 2A and S2 Fig). The analysis further suggested a M9 peptidase domain (aa 93–634), a PKD domain (aa 770–852) and a prepeptidase c-terminal (PPC) domain (aa 880–947), which corresponds to the CBD domain of ColG (S2 Fig).

According to the predicted domain architecture, *B*. *cereus* ATCC 14579 ColA variants lacking the putative signal peptide (ColA ΔSP) or also the predicted propeptide (ColA ΔPP) were cloned and overexpressed in *E*. *coli* to purify ColA ΔSP and ColA ΔPP as N-terminally tagged GST-ColA fusion proteins (Fig 2A). Lysates of *E*. *coli* transformed with expression constructs for GST-ColA ΔSP^wt^ were analyzed by coomassie-stained SDS PAGE (S3A Fig, lane 1). GST-ColA ΔSP^wt^, which had a predicted molecular weight of approximately 132.8 kDa was not observed, but appeared to be processed into a ~100 kDa protein and a 26 kDa GST protein as detected by SDS PAGE (S3A Fig). The identity of GST was also confirmed by Western blotting (S3A Fig, lanes 9–12). To analyze whether an autoproteolytic processing of GST-ColA ΔSP^wt^ occurred, a protease-inactive variant was created by the exchange of the glutamic acid 501 to an alanine in the active center (ColA ΔSP^E501A^). In contrast to GST-ColA ΔSP^wt^ in bacterial lysates, proteolytic-inactive GST-ColA ΔSP^E501A^ migrated with the predicted molecular weight of approximately 132.8 kDa (S3A Fig, lane 2). In GST pull-down (PD) experiments with GST-ColA ΔSP^wt^, only the 26 kDa GST tag could be enriched, while the GST-ColA ΔSP^E501A^ samples yielded the 132.8 kDa full length protein (S3A Fig, lanes 3–4). Furthermore, the gelatinolytic activities of these proteins were analyzed by zymography. GST-ColA ΔSP is gelatinolytically active, but exhibited a lower molecular weight in the range of 100 kDa than the expected 132.8 kDa (S3A Fig, lane 5, asterisk). The GST-ColA ΔSP^E501A^ mutant was proteolytically inactive (S3A Fig, lanes 6 and 8). Together, these data indicate that the N-terminal GST tag of ColA ΔSP was autocatalytically removed.

Subsequently, we generated isogenic GST-ColA ΔPP^wt^ and its inactive GST-ColA ΔPP^E501A^ mutant for the further characterization of recombinant *B*. *cereus* ATCC 14579 ColA (Fig 2A and 2B). GST-tagged proteins lacking the propeptide exhibited the predicted molecular weight of 125.6 kDa and were detected in the lysates of IPTG-induced *E*. *coli* (Fig 2B, lanes 1–2) and in GST-PD experiments (Fig 2B, lanes 3–4). Comparing the molecular weight of autoprocessed GST-ColA ΔSP^wt^ in *E*. *coli* lysates (S3B Fig, lane 7) with the different recombinant protein variants GST-ColA ΔSP^E501A^ (132.8 kDa), ColA ΔSP^E501A^ (106.8 kDa), GST-ColA ΔPP^wt^ (125.6 kDa), ColA ΔPP^wt^ (99.6 kDa), GST-ColA ΔPP^E501A^ (125.6 kDa) and ColA ΔPP^E501A^ (99.6 kDa) (S3B Fig) showed that the autoprocessed GST-ColA ΔSP^wt^ migrated at the size of the 106.8 kDa ColA ΔSP^E501A^ protein. This indicated that ColA^wt^ cleaves off the GST tag, but not the propeptide and could further indicate that ColA cleaves-off its signal peptide in an autocatalytic manner. Therefore, we aimed to identify the cleavage site by mass-spectrometry analyses of the processed GST tag. These analyses revealed that ColA cleaves in the amino acid stretch FQ^↓^GPL in the linker between the GST tag and ColA ΔSP^wt^ protein (S4 Fig).

For further analyses of the proteolytic activity of ColA, we finally expressed GST-ColA ΔPP^wt^ protein in *E*. *coli*. In contrast to the inactive GST-ColA ΔPP^E501A^ mutant (Fig 2B, lanes 6 and 8), GST-ColA ΔPP^wt^ proteins were gelatinolytic active in zymography analyses (Fig 2B, lanes 5 and 7). Both, induction and enrichment of GST-ColA ΔPP^wt^ protein were analyzed by coomassie-stained SDS PAGEs (Fig 2B, lanes 9–11). To remove the GST tag from the fusion protein, GST-ColA ΔPP^wt^ coupled to GST sepharose was incubated with PreScission protease resulting in the release of ColA ΔPP^wt^ (Fig 2B, lane 12).

### Recombinant *B*. *cereus* ATCC 14579 ColA Acts as a True Collagenase

**Fig 3:**
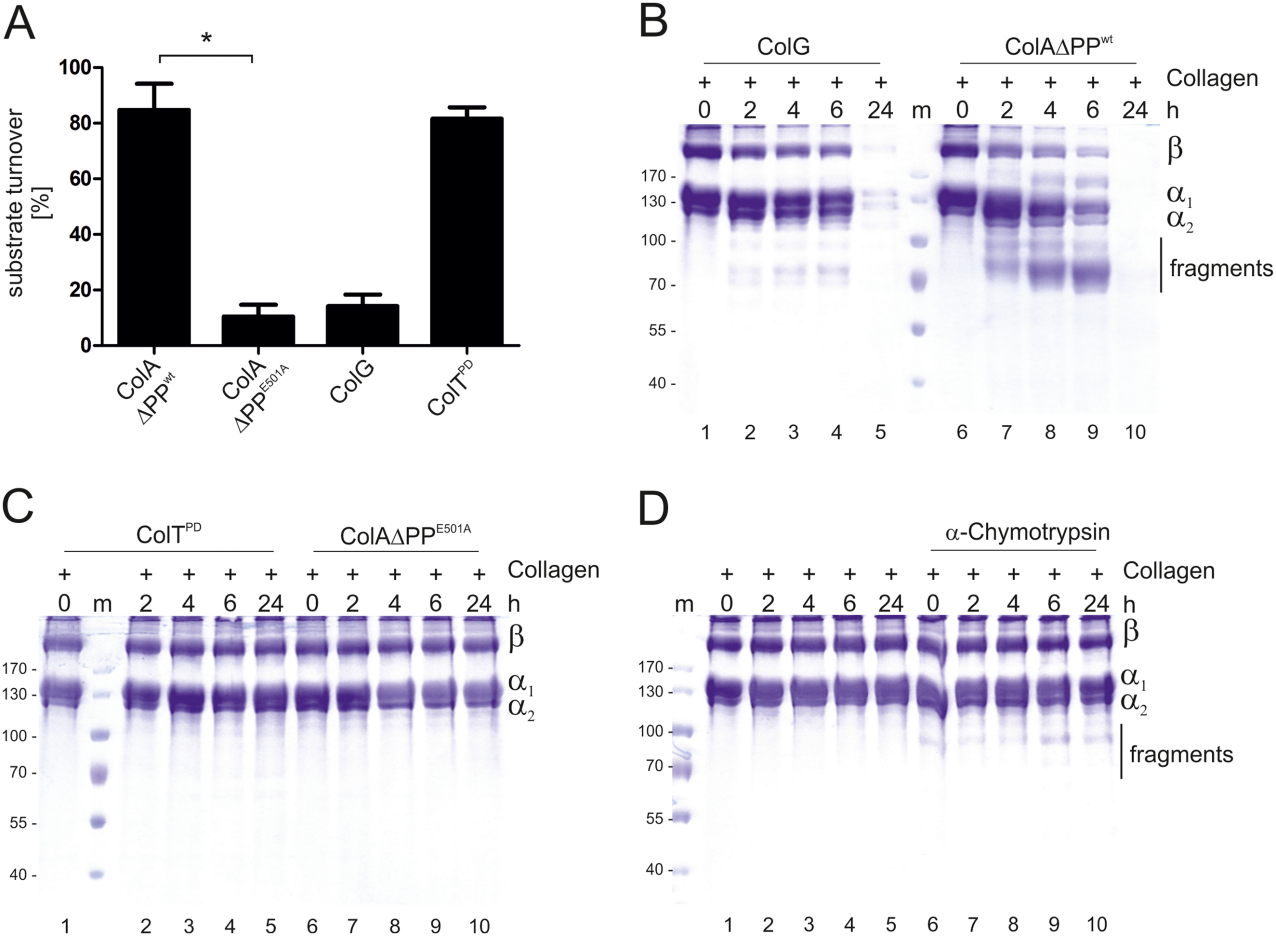
Collagenolytic activity of ColA. (**A**) ColA ΔPP from *B*. *cereus* ATCC 14579, its inactive version ColA ΔPP^E501A^, ColG from *C*. *histolyticum* and the protease domain of ColT (ColT^PD^) from *C*. *tetani* were tested for their peptidase activity using FALGPA as a substrate. * *p* = 0.0161 indicates statistical significance (Student´s t-test, paired, one-tailed). (**B**) In *in vitro* cleavage assays, the positive control ColG and ColA ΔPP^wt^ were incubated with tropocollagen type I for the indicated time periods and analyzed by coomassie stained SDS PAGE to analyze their collagenolytic activities. (**C**) As negative controls, ColT^PD^ and ColAΔPP^E501A^ were investigated. (**D**) As indicated, α–chymotrypsin was incubated with tropocollagen type I as an additional negative control and compared to untreated tropocollagen type I for the indicated time periods and analyzed by coomassie stained SDS PAGE.

To examine the peptidase activity of recombinant ColA ΔPP^wt^ from *B*. *cereus* ATCC 14579 in more detail, N-[3-(2-Furyl)acryloyl]-Leu-Gly-Pro-Ala (FALGPA) was used as a synthetic peptide-substrate mimicking the consensus collagenase cleavage site. We compared the activity of the *B*. *cereus* ATCC 14579 ColA ΔPP^wt^ with the recombinant protease domain of ColT from *C*. *tetani* (ColT^PD^) [12]. Proteolytic inactive ColA ΔPP^E501A^ and ColG from *C*. *histolyticum* exhibiting low peptidolytic activity [12] were included as negative controls. Incubation of FALGPA with ColA ΔPP^wt^ led to a rapid substrate turnover of ~85% after 12 min, which was comparable with the activity of ColT^PD^ (Fig 3A).

To further analyze the collagenolytic activity, we tested collagenases on their ability to cleave tropocollagen I composed of the β, α_1_ and α_2_ chains (Fig 3B, lanes 1 and 6). Incubation of tropocollagen with ColG induced a slight fragmentation of tropocollagen after 6 h, which drastically increased after 24 h (Fig 3B, lanes 2–5). A strong fragmentation of tropocollagen was observed after incubation of tropocollagen with ColA ΔPP^wt^ as no collagen was detectable after 24 h (Fig 3B, lanes 7–10). This underlines that ColA from *B*. *cereus* ATCC 14579 targets not only denatured collagen, but efficiently cleaves tropocollagen and thus can be considered as a true collagenase. Most likely, ColA is the main collagenolytic entity described in *B*. *cereus* supernatants [17]. In contrast, the peptidase domain of ColT^PD^ which lacked the activator domain, and the inactive ColA ΔPP^E501A^ did not cleave tropocollagen (Fig 3C), similar to chymotrypsin which only induced a limited cleavage of collagen (Fig 3D). These data underline the finding that ColA expressed by *B*. *cereus* ATCC 14579 can target native collagen. ColA activity appeared highly specific as we did not identify further substrates from the ECM, such as laminin and fibrinogen (S5A Fig) or vitronectin (S5B Fig). Casein was included as an additional protease substrate (S5B Fig).

**Fig 4:**
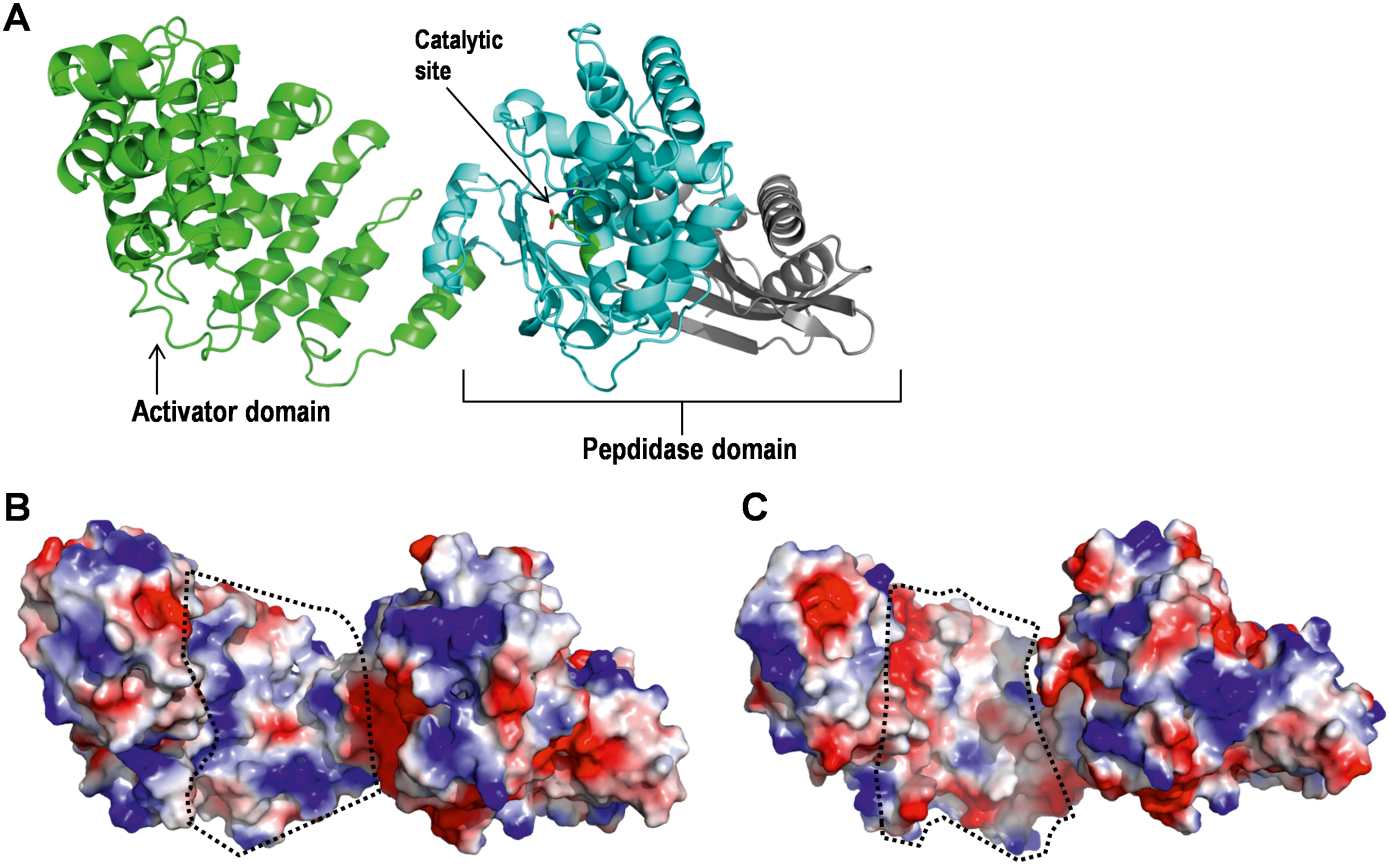
Homology model of *B*. *cereus* ATCC 14579 ColA and the comparison of the surface property of ColA and ColG from *C*. *histolyticum* near the catalytic site. (**A**) Ribbon representation of modeled collagenase module of *B*. *cereus* ATCC 14579 ColA, which consists of an N-terminal activator domain (green) followed by a catalytic subdomain (cyan) and a C-terminal catalytic helper subdomain (grey). Electrostatic surface potential of collagenase module of (**B**) *B*. *cereus* ATCC 14579 ColA and (**C**) *C*. *histolyticum* ColG (PDB 2Y3U). The basic and acidic regions are shown in blue and red, respectively.

In comparison with ColG, *B*. *cereus* ATCC 14579 ColA is exceptionally active. However, a crystal structure of *B*. *cereus* ATCC 14579 ColA is not available yet, which could explain the increased activity. Therefore, comparative protein modelling was used to model the residues Y^93^-K^850^ of *B*. *cereus* ATCC 14579 ColA, which comprises the activator domain, a catalytic subdomain and a catalytic helper subdomain (Fig 4A). The quality of the obtained model (Y^93^-K^850^) was evaluated by RAMPAGE [40] and 95.8% of the modeled residues are present in the favored region, 3.1% of them are in the allowed region and 1.0% residues are outliers. Based on this homology model, the electrostatic surface potential of *B*. *cereus* ATCC 14579 ColA was calculated and compared to ColG (Fig 4B and 4C). Interestingly, this analysis revealed that the surface properties of ColA and ColG at the activator domain (Fig 4B and 4C, dotted lines) differed significantly. ColA exhibited a highly basic surface, whereas the corresponding region in ColG was acidic, which might potentially explain the difference in their activities.

### *B*. *cereus* ATCC 14579 ColA Expression Is Under Control of the plcR Regulon

**Fig 5:**
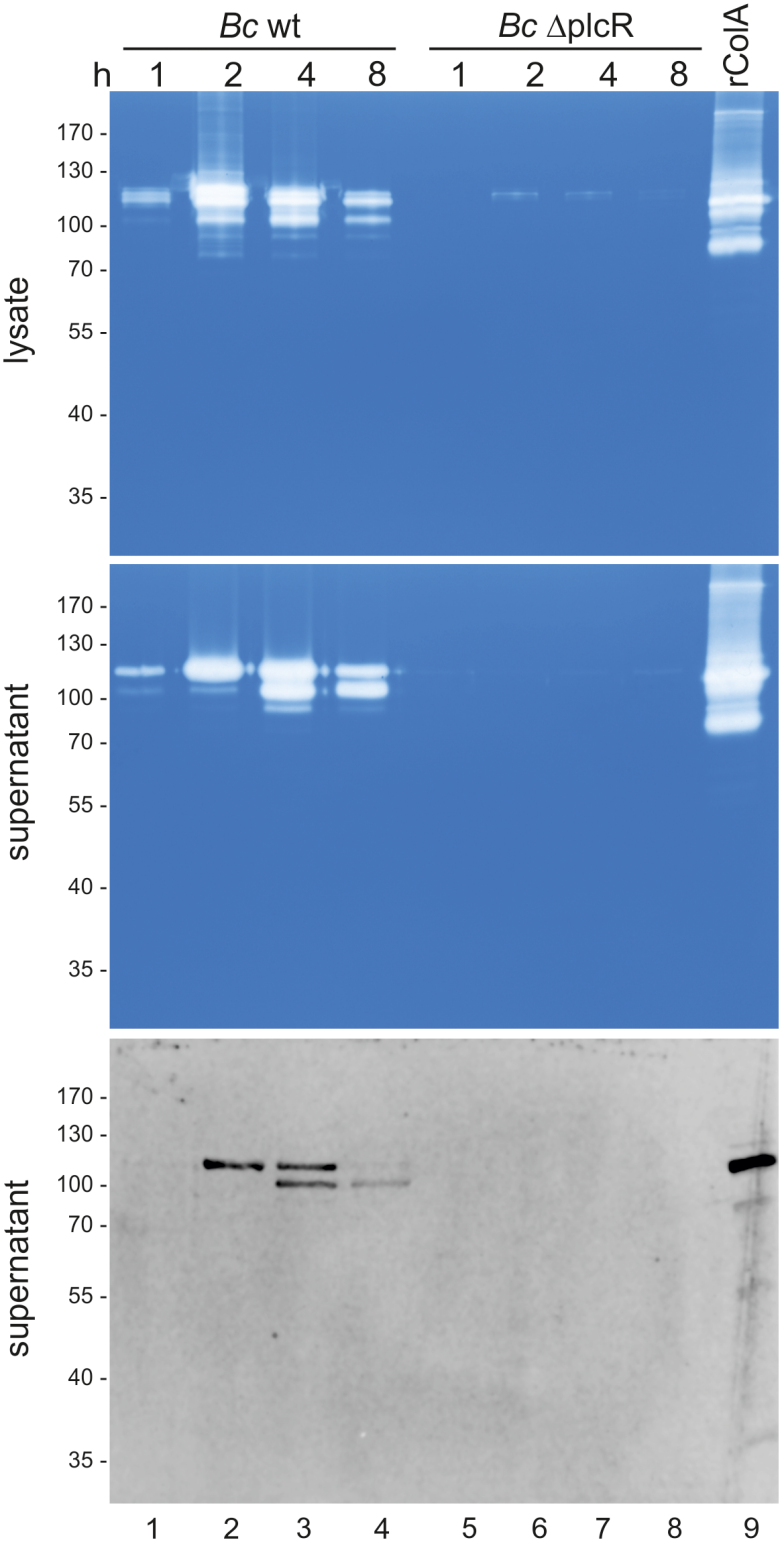
ColA is secreted by *B*. *cereus* ATCC 14579. *B*. *cereus* ATCC 14579 wildtype (wt) and its isogenic *ΔplcR* deletion mutant were harvested and disrupted after growing in liquid cultures for indicated time periods. Equal protein amounts of bacterial lysates (upper panel) and equal volumes of supernatants were analyzed by gelatin zymography (upper and middle panel) and Western blotting using a polyclonal antibody directed against ColA ΔPP^E501A^ (lower panel). Recombinant ColA ΔPP (rColA) was used as control.

Based on the finding that both endogenous and recombinant ColA are highly active and true collagenases, we analyzed the activities of collagenases in bacterial lysates and supernatants of *B*. *cereus* ATCC 14579 wildtype (wt) and an isogenic *plcR* deletion mutant (ΔplcR). *B*. *cereus* ATCC 14579 strains were cultured in BHI medium and after indicated time periods, equal amounts of bacterial lysates and supernatants were analyzed for collagenase activity in gelatin zymography. As a control, recombinant ColA ΔPP^wt^ (rColA) was included (Fig 5, lane 9). In fact, we observed several gelatinolytic activities in the lysates of *B*. *cereus* ATCC 14579 wt (Fig 5, lanes 1–4, upper panel). To correlate the observed activity with ColA expression, we generated a polyclonal anti-ColA antibody raised against rColA (ColA **Δ**PP^E501A^). We detected ColA protein in bacterial lysates in Western blot analysis (S6 Fig), which corresponds to gelatinolytic activities (Fig 5, upper panel). ColA activity was increased at 2 h and 4 h and decreased again at 8 h post-inoculation. Only a weak activity was observed in the *plcR* deletion mutant (Fig 5, lanes 5–8, upper panel) indicating that ColA is regulated by the plcR regulon as described previously [31, 33]. Collagenase activities appeared at least at four different molecular weights suggesting that multiple ColA variants were present (Fig 5, upper panel). The intensity of the individual proteolytic activities was slightly changed in the culture supernatants at different growth phases (Fig 5, middle panel). Here, we detected three main activities, which increased at 2 h and 4 h of culture. The activity with the highest molecular weight decreased at 8 h, while the activity of intermediate size increased (Fig 5, lanes 1–4, middle panel). The identity of secreted ColA was further validated by Western blot analysis (Fig 5, lower panel). Our data suggest that ColA processing occurs during secretion, which could be substantiated by the detection of different ColA versions in Western blot analyses (Fig 5, lower panel). In comparison with 99.6 kDa rColA, the largest secreted ColA version from *B*. *cereus* ATCC 14579 migrates at the same molecular weight (Fig 5, lanes 1–4). Putative cleavage products were produced, which can result from N-terminally, but also from C-terminal processing as described for ColG [44].

## Discussion

Bacterial proteases are often directly or indirectly related to their virulence as they can either interfere directly with host cell functions or are involved in the processing of other bacterial virulence factors [45, 46]. Collagenases attracted special attention since they can hydrolyze collagen, which represents an important structural component of the ECM. For instance, the collagenase of *V*. *vulnificus* has been described to promote bacterial invasion into human tissue [47, 48]. *Helicobacter pylori* expresses the collagenase Hp0169, which was demonstrated to be an essential factor for bacterial colonization of the murine stomach [49]. Similar roles have been suggested for clostridial collagenases indicating the importance of bacterial collagenases in microbial pathogenesis [7, 8]. In the last decades, clostridial collagenases also have been applied in safe and efficient therapeutic intervention strategies for Peyronie's or Dupuytren diseases [50, 51]. Hence, intensive research on clostridial proteases increased the knowledge on structure, activity and function of collagenases. However, less is known about collagenases expressed by *B*. *cereus s*.*l*. In our previous work, we observed a highly active collagenase expressed by *B*. *cereus* ATCC 14579 [35], which has been reported as a collagenase in the supernatants of *B*. *cereus s*.*s*. in earlier studies [17, 18], but was not further characterized. In this study we provide evidence that the described gelatinolytic activity is the true collagenase ColA secreted by *B*. *cereus* ATCC 14579, and which targets helical collagen.

Collagenases from Clostridia were described as zinc-dependent metalloproteases composed of an N-terminal signal peptide, a putative propeptide, an activator domain followed by the catalytic peptidase domain, and a varying number of PKD and CBD domains [8, 9]. Clostridial domain architecture and structure have been intensively investigated revealing the molecular mechanisms of true collagenases [11–13]. Corresponding to previous reports [17, 18], ColA also acts as a true collagenase, which targets gelatin, FALGPA and native tropocollagen type I, indicating that ColA prefers the typical collagen motifs Gly-Pro-X and Gly-X-Hyp [14] as cleavage sites. In comparison with the collagenase paradigm ColG from *C*. *histolyticum*, *B*. *cereus* ATCC 14579 ColA exhibited a fast enzymatic degradation of tropocollagen. Analyzing structural differences, a homology model of the collagenase activator and peptidase domains of *B*. *cereus* ColA points to alterations of the surface charge of ColA and ColG that could possibly explain the different enzymatic activities. This feature might play a significant role in collagen recognition and binding and could help to explain the difference in the activities of these two proteases.

*B*. *cereus* collagenase has previously been described as a secreted protease [17]. Accordingly, we mainly observed two proteolytic activities in supernatants of *B*. *cereus* ATCC 14579. Although we cannot exclude the possibility that additional collagenases were expressed in *B*. *cereus*, our data suggest that ColA processing occurs during secretion, which could be substantiated by the detection of different ColA variants in Western blot analyses. Since the size of secreted ColA matched the size of recombinant ColA ΔPP, we assume that secreted ColA lacks not only the signal peptide, but also the propeptide. As the maturation of ColA was not investigated in this study, it remains unknown if ColA is a self-processing protease or if additional proteases are implicated in ColA secretion. In fact, several functions have been proposed for N-terminally located signal peptides and propeptides in Gram-positive and Gram-negative bacteria. It was suggested that propeptides are required for proper folding, for secretion of the mature protease, for maintaining the protease in an inactive state, or for anchoring the protease to the membrane [52]. N-terminal cleavage and conversion from the proform of the protease into an active form was already described for other well characterized metalloproteases like for MMP´s [53] and for trypsin-like proteases [54]. For the metalloprotease Npr of *Streptomyces cacaoi*, it has been proposed that the signal peptide directs Npr to the membrane. After removal of the signal peptide, Npr is released into the environment, where the propeptide is cleaved-off [55, 56]. According to our data, the secreted *B*. *cereus* ColA did not contain the propeptide, therefore we assume that the removal of the signal peptide and propeptide is involved in ColA secretion via a yet unknown mechanism. Additional processing of ColA in bacterial supernatants was observed, which could reflect C-terminal cleavage events as already proposed for other collagenases [7, 13, 57]. However, the molecular consequences of ColA cleavage on the protease function needs to be investigated in future studies.

Many virulence factors of *B*. *cereus* are controlled by the transcriptional regulator PlcR [31] via binding to the PlcR box upstream of regulated target genes [32, 33]. Described as a pleiotropic regulator, PlcR can control the expression of a wide range of virulence-associated genes including enterotoxins, cytotoxins, and hemolysins [33]. Here, we observed that ColA expression is down-regulated in a *plcR*-negative *B*. *cereus* mutant, which is in line with a previous study that mapped a PlcR box upstream of the *bc3161* gene in the *B*. *cereus* ATCC 14579 strain [33]. Transcription of PlcR is increased in bacteria at the beginning of the stationary growth phase [58], which correlates with PlcR-controlled expression of secreted *B*. *cereus* factors [59]. This is in contrast to another report suggesting a decrease of the *plcR* transcript and PlcR-controlled *nhe* and *hbl* expression in the stationary growth phase of the anaerobic *B*. *cereus* F4430/73 [60]. Although we did not analyze the PlcR expression in *B*. *cereus* ATCC 14579 in our experiments under aerobic conditions, we conclude that PlcR-dependent ColA expression and secretion already start in the logarithmic growth phase [35].

In conclusion, we identified ColA as the major secreted true collagenase of *B*. *cereus* ATCC 14579. The existence of additional orthologs to the *colA* gene *bc_3161* in other sequenced *B*. *cereus sensu stricto* strains as well as *B*. *cereus* group strains (Table 2) implies that expression of bacilli collagenase is a wide spread phenomenon and needs to be investigated in future. Cloning and purification of recombinant ColA revealed that it is a highly active protease that efficiently targets native tropocollagen. Furthermore, it might contribute to bacterial virulence of *B*. *cereus* in endophthalmitis or opportunistic infections via collagen degradation in the ECM. Clostridial collagenases already represent a widespread compound in the treatment of several diseases, such as Peyronie's or Dupuytren diseases. The high activity of *B*. *cereus* ColA makes it an additional attractive candidate for medical treatments.

## Supporting Information

S1 Fig Alignment of ColT from *B*. *cereus* m1293 and ColA from *B*. *cereus* ATCC 14579. Protein sequences from *B*. *cereus* m1293 ColT and B. cereus Bc3161 were retrieved from UniProt. Sequence alignments were performed using Clustal Omega. (*) indicates identical amino acids in all sequences, conserved amino acid substitutions are labeled with (:) and semi-conservative substitutions are marked with (.).
(TIF)

S2 Fig Alignment of ColA from *B*. *cereus*, ColH from *C*. *histolyticum* and ColG from *C*. *histolyticum*. ColG from *C*. *histolyticum* harbors an activator domain (green), peptidase domain (blue), a PKD domain (yellow), and the two CBD domains alpha (grey), and beta (dark grey). ColH harbors a second PKD domain, but only one CBD domain. Sequence analysis revealed that ColA from *B*. *cereus* contains a signal peptide (aa 1–30, black box) and predicted a propeptide in the N-terminus of ColA (aa 31–92, red box). A M9 peptidase domain (aa 93–634), is followed by a PKD domain (aa 770–852) and a prepeptidase c-terminal (PPC) or CBD domain (aa 880–947).
(TIF)

S3 Fig Expression of ColA ΔSP and ColA ΔPP proteins. (**A**) GST-ColA ΔSP^wt^ and GST-ColA ΔSP^E501A^ proteins in lysates or precipitated by GST pull down (PD) experiments were analyzed by SDS-PAGE (left panel), by gelatin zymography (middle panel), and by Western blot analysis using a polyclonal antibody directed against GST (right panel). (**B**) 1 μg of tested proteins after GST-PD experiments was separated by SDS PAGE and stained using coomassie. Where indicated the GST tag was removed by the PreScission protease. The protein standard (m) shows the actual size of proteins in the coomassie-stained gel (on the left) in comparison to the theoretical molecular weight as indicated on the right (lanes 1–8). Lysates of induced *E*. *coli* transformed with the expression plasmid encoding the GST-ColA ΔSP^wt^ were included to verify the molecular weight of the processed protein (lane 9).
(TIF)

S4 Fig Intact mass spectrum of reduced GST-ColA ΔSP (a) and GST fragment (b). Mass spectrometry revealed that the cleavage site is C-terminal to Q226 at the LFQ/GPL motif. Deconvolution of the intact ion spectra was carried out with the Xtract algorithm integrated into the software Xcalibur 3.0.63 (Thermo Fisher Scientific).
(TIF)

S5 Fig Substrate selectivity of ColA. The putative substrates laminin (1 μg), fibrinogen (1 μg), collagen (1 μg) (**A**) or vitronectin (1 μg) and casein (5 μg) (**B**) were incubated with 1 μg ColA or left untreated (-) for 16 hours. Proteins were separated by SDS PAGE followed by coomassie staining.
(TIF)

S6 Fig Detection of ColA in the lysates of *B*. *cereus*. Equal protein amounts of bacterial lysates of *B*. *cereus* ATCC 14579 grown for indicated time periods were analyzed by Western blotting. The polyclonal anti-ColA antibody detected endogenous ColA. An unspecific cross-reactivity has been indicated by an asterisk (*). Recombinant ColA ΔPP^wt^ (rColA) served as a control.
(TIF)
