# Supplementary figures and images for: Cloning, Purification and Characterization of the Collagenase ColA Expressed by Bacillus cereus ATCC 14579
Source: PLoS One. 2016 Sep 2;11(9):e0162433. doi: 10.1371/journal.pone.0162433 (PMC5010206; doi:10.1371/journal.pone.0162433)

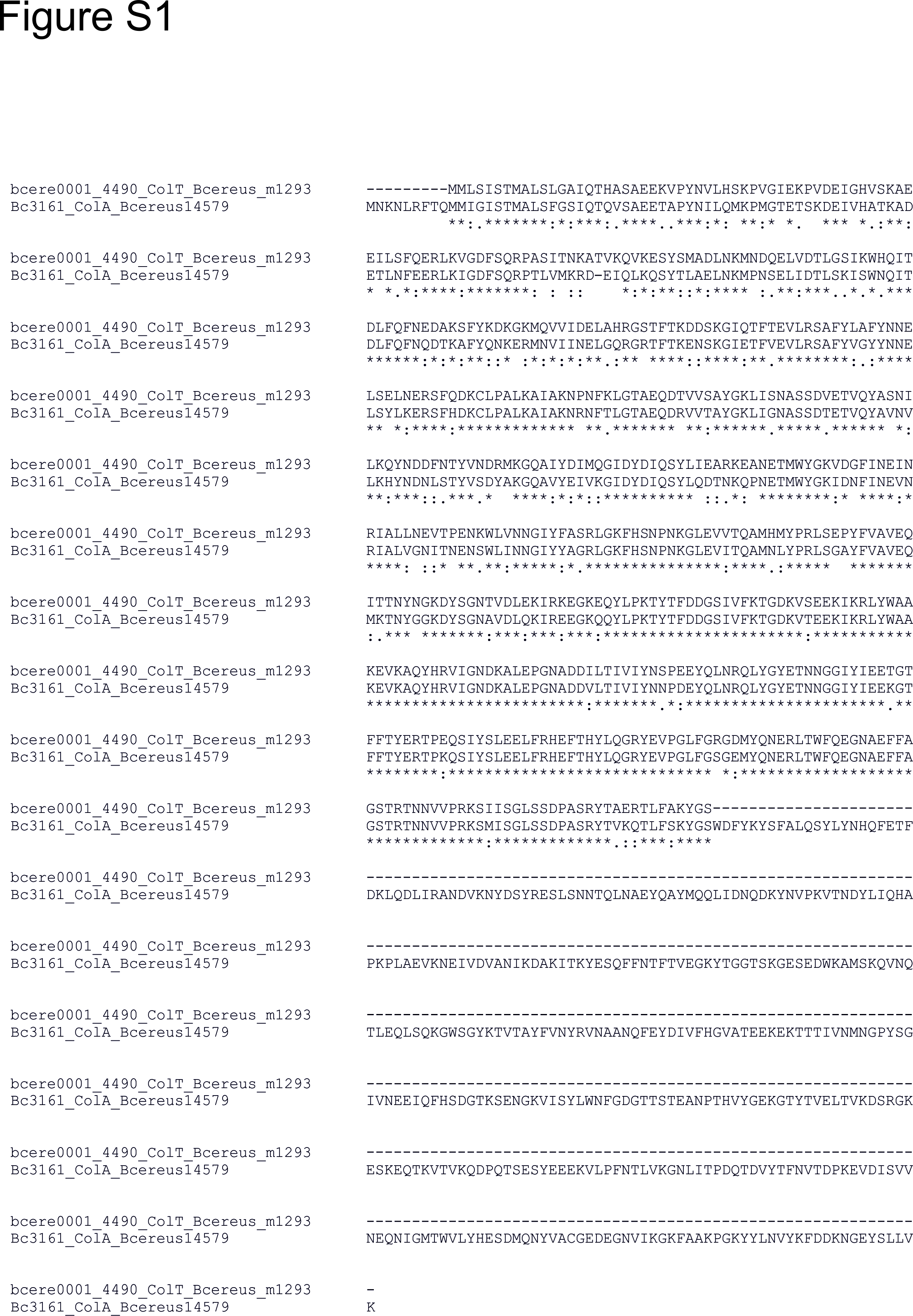

Supplement: S1 Fig — Protein sequences from B. cereus m1293 ColT and B. cereus Bc3161 were retrieved from UniProt. Sequence alignments were performed using Clustal Omega. (*) indicates identical amino acids in all sequences, conserved amino acid substitutions are labeled with (:) and semi-conservative substitutions are marked with (.). (TIF) [file pone.0162433.s001.tif]

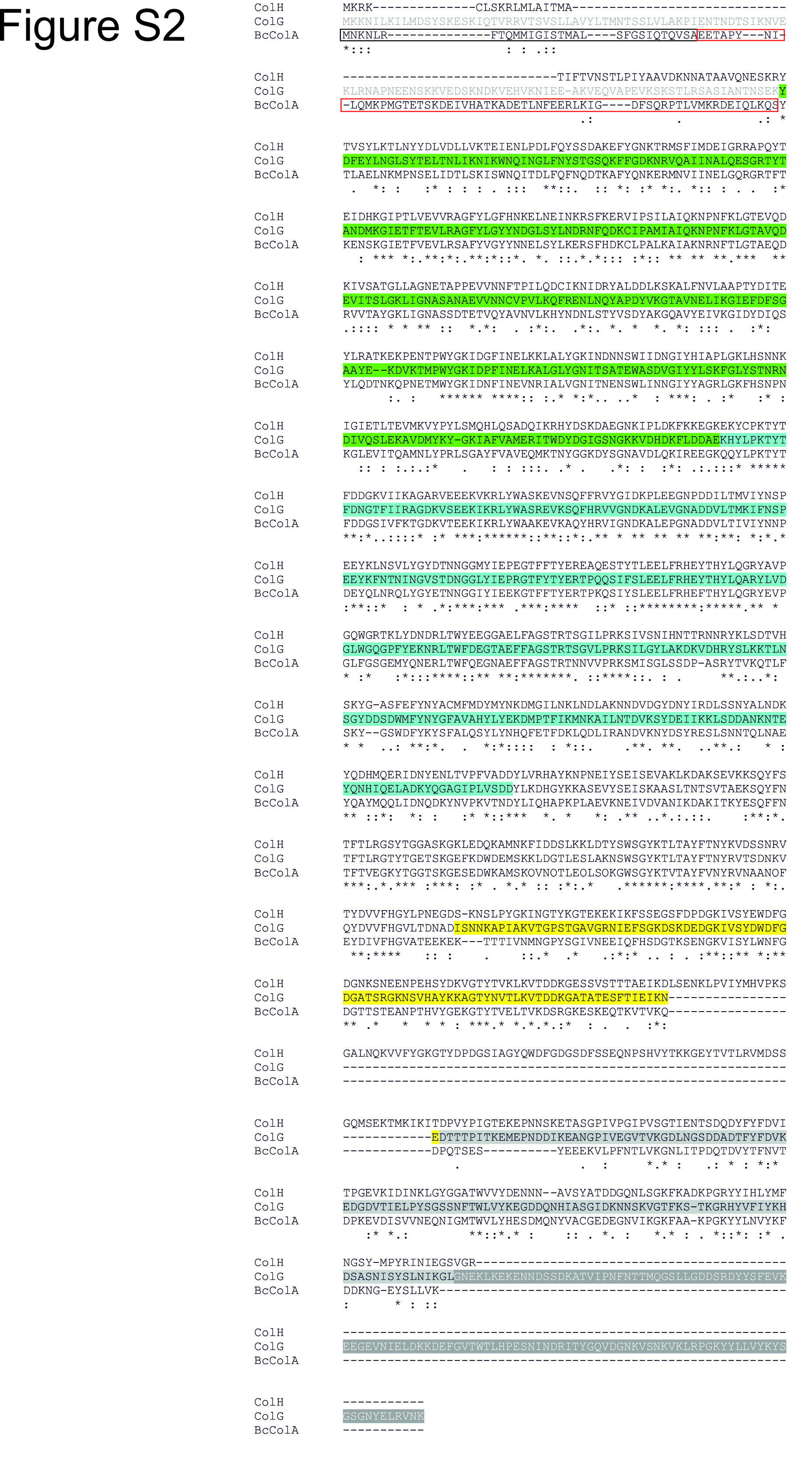

Supplement: S2 Fig — ColG from C. histolyticum harbors an activator domain (green), peptidase domain (blue), a PKD domain (yellow), and the two CBD domains alpha (grey), and beta (dark grey). ColH harbors a second PKD domain, but only one CBD domain. Sequence analysis revealed that ColA from B. cereus contains a signal peptide (aa 1–30, black box) and predicted a propeptide in the N-terminus of ColA (aa 31–92, red box). A M9 peptidase domain (aa 93–634), is followed by a PKD domain (aa 770–852) and a prepeptidase c-terminal (PPC) or CBD domain (aa 880–947). (TIF) [file pone.0162433.s002.tif]

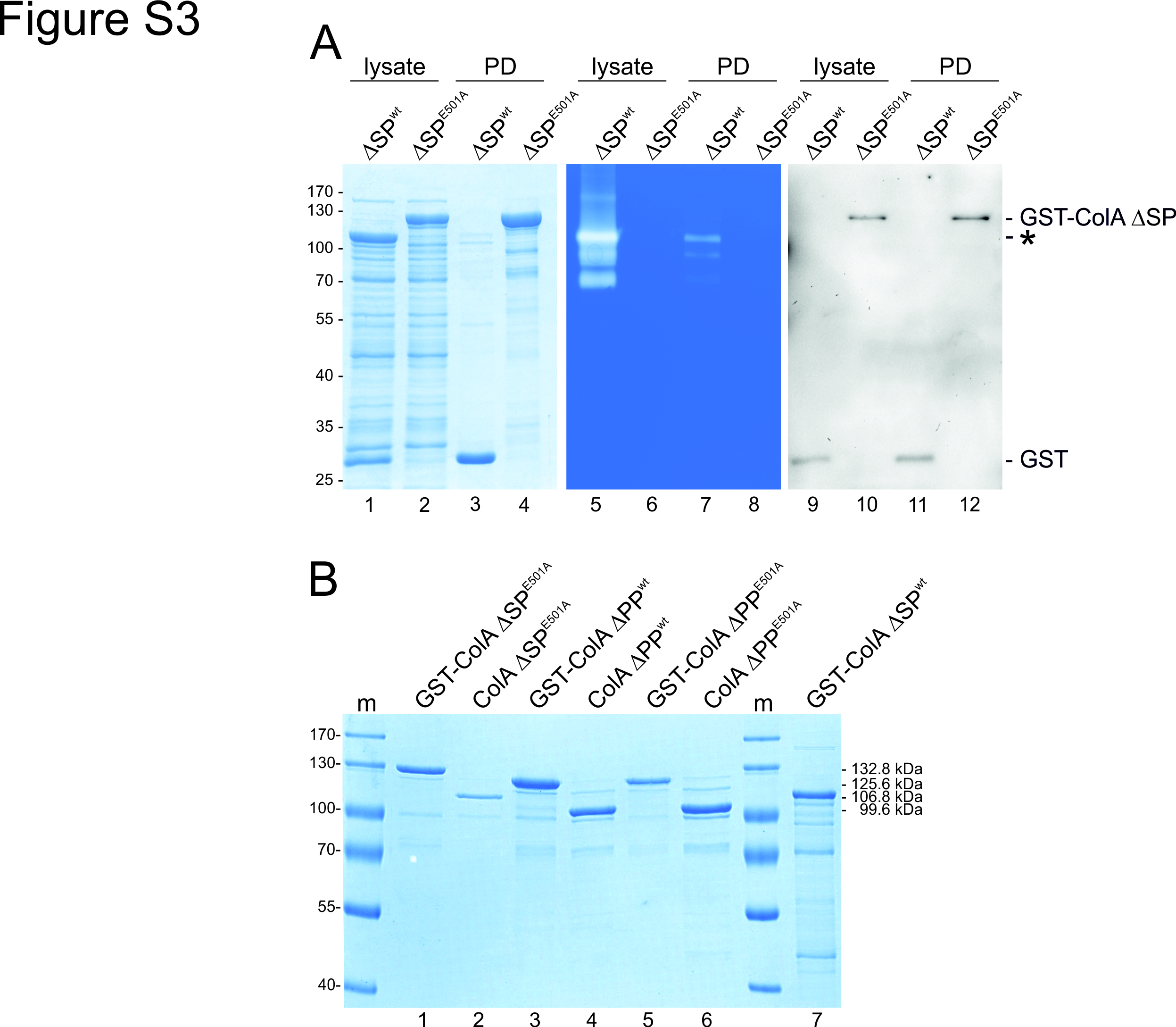

Supplement: S3 Fig — (A) GST-ColA ΔSPwt and GST-ColA ΔSPE501A proteins in lysates or precipitated by GST pull down (PD) experiments were analyzed by SDS-PAGE (left panel), by gelatin zymography (middle panel), and by Western blot analysis using a polyclonal antibody directed against GST (right panel). (B) 1 μg of tested proteins after GST-PD experiments was separated by SDS PAGE and stained using coomassie. Where indicated the GST tag was removed by the PreScission protease. The protein standard (m) shows the actual size of proteins in the coomassie-stained gel (on the left) in comparison to the theoretical molecular weight as indicated on the right (lanes 1–8). Lysates of induced E. coli transformed with the expression plasmid encoding the GST-ColA ΔSPwt were included to verify the molecular weight of the processed protein (lane 9). (TIF) [file pone.0162433.s003.tif]

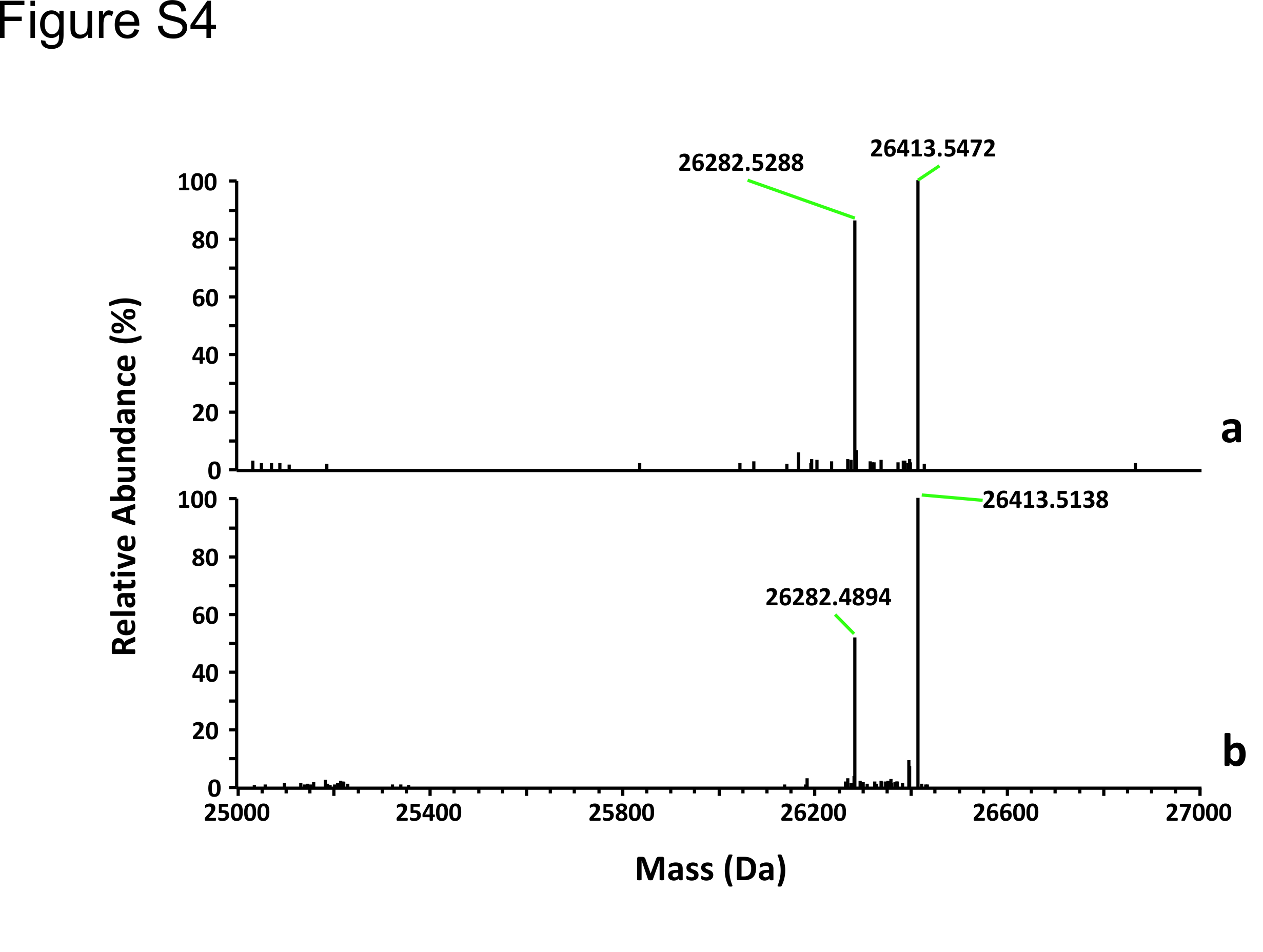

Supplement: S4 Fig — Mass spectrometry revealed that the cleavage site is C-terminal to Q226 at the LFQ/GPL motif. Deconvolution of the intact ion spectra was carried out with the Xtract algorithm integrated into the software Xcalibur 3.0.63 (Thermo Fisher Scientific). (TIF) [file pone.0162433.s004.tif]

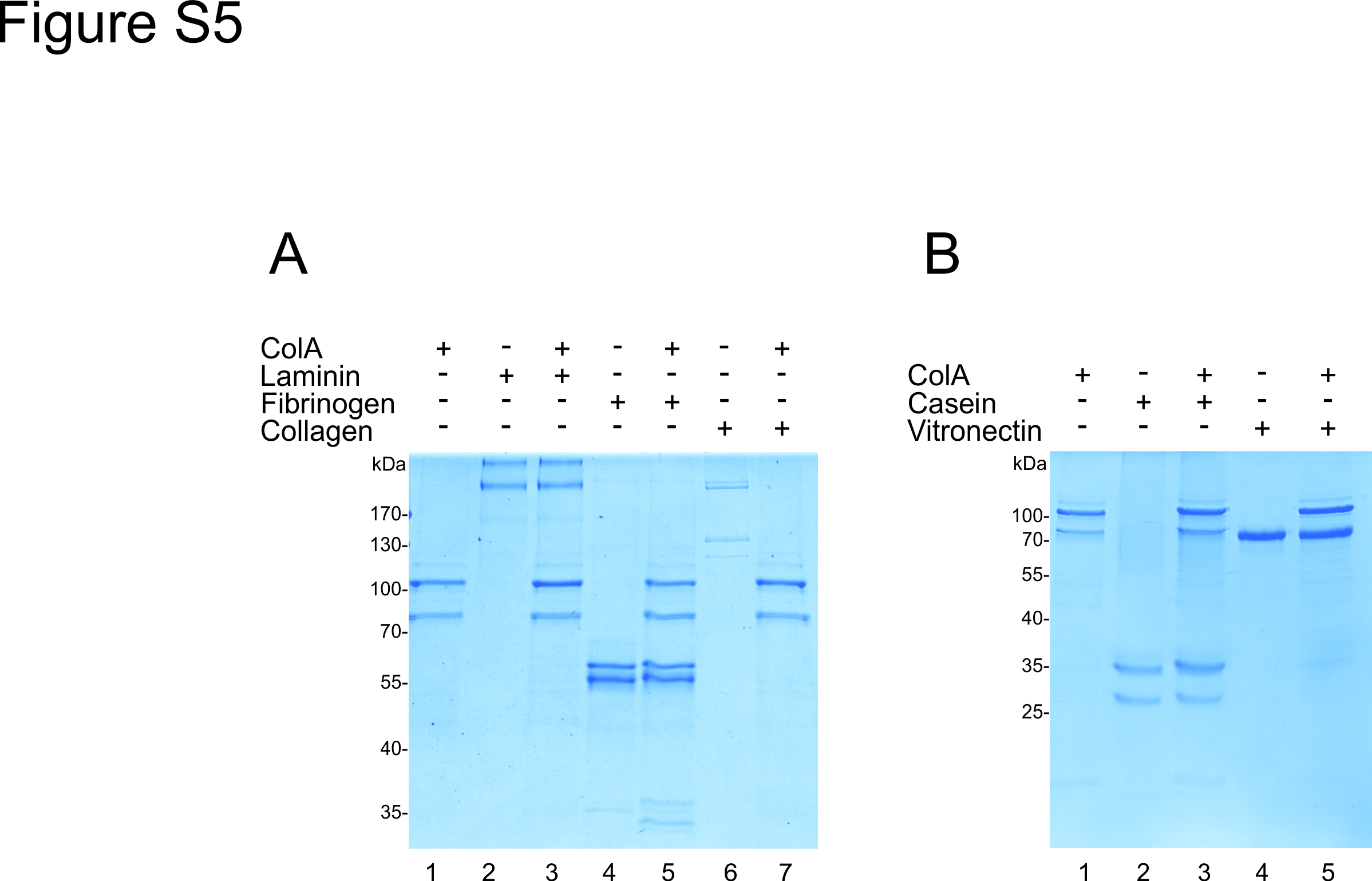

Supplement: S5 Fig — The putative substrates laminin (1 μg), fibrinogen (1 μg), collagen (1 μg) (A) or vitronectin (1 μg) and casein (5 μg) (B) were incubated with 1 μg ColA or left untreated (-) for 16 hours. Proteins were separated by SDS PAGE followed by coomassie staining. (TIF) [file pone.0162433.s005.tif]

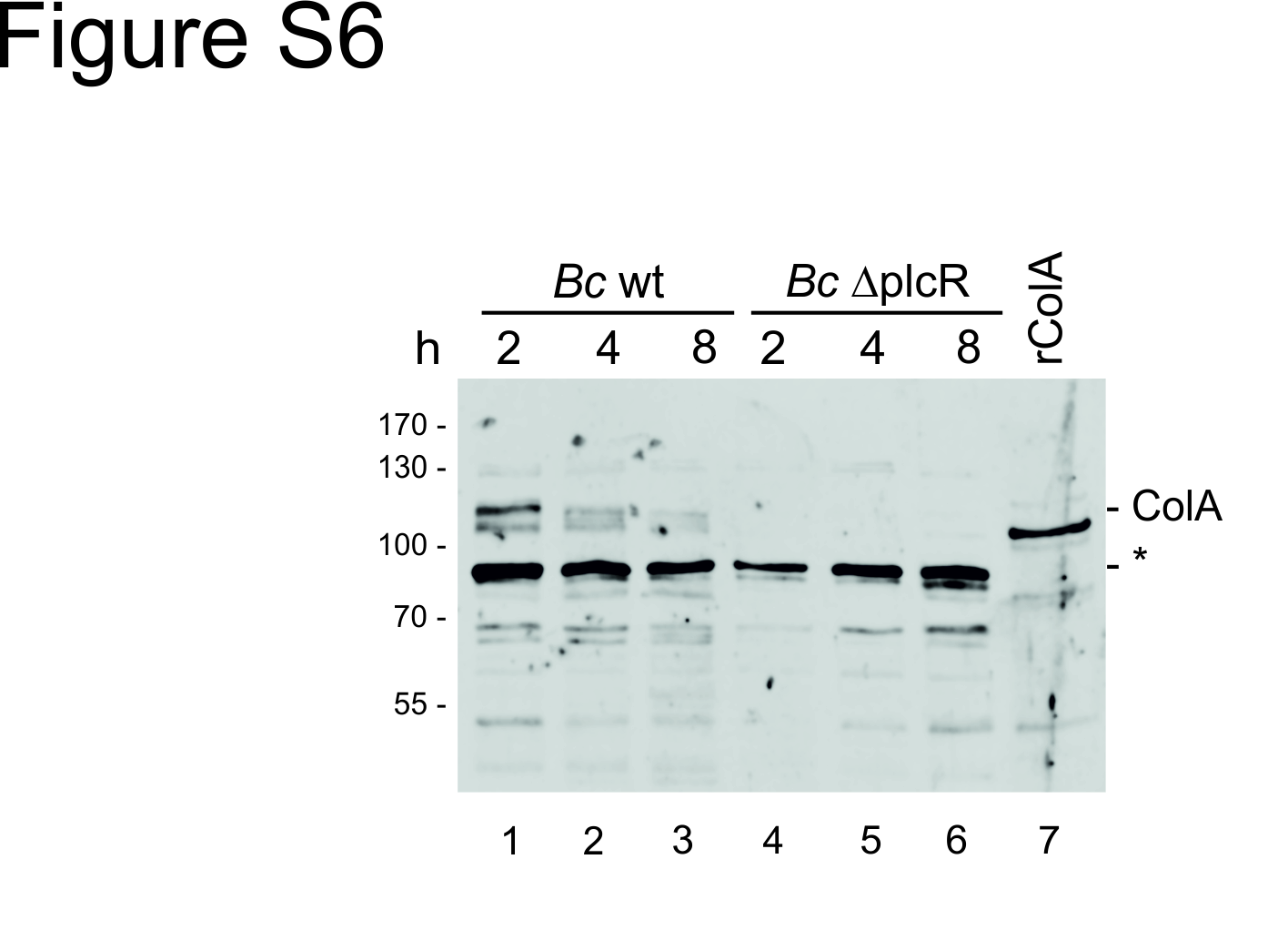

Supplement: S6 Fig — Equal protein amounts of bacterial lysates of B. cereus ATCC 14579 grown for indicated time periods were analyzed by Western blotting. The polyclonal anti-ColA antibody detected endogenous ColA. An unspecific cross-reactivity has been indicated by an asterisk (*). Recombinant ColA ΔPPwt (rColA) served as a control. (TIF) [file pone.0162433.s006.tif]
